# Class II malocclusion occlusal severity description

**DOI:** 10.1590/S1678-77572010000400013

**Published:** 2010

**Authors:** Guilherme JANSON, Renata SATHLER, Thais Maria Freire FERNANDES, Marcelo ZANDA, Arnaldo PINZAN

**Affiliations:** 1 DDS, MSc, PhD, MRCDC (Member of the Royal College of Dentists of Canada), Professor and Head, Department of Pediatric Dentistry, Orthodontics and Community Health, Bauru School of Dentistry, University of São Paulo, Bauru, SP, Brazil.; 2 DDS, Orthodontics Graduate Student, Department of Pediatric Dentistry, Orthodontics and Community Health, Bauru School of Dentistry, University of São Paulo, Bauru, SP, Brazil.; 3 DDS, MSc, PhD, Department of Stomatology, Bauru School of Dentistry, University of São Paulo, Bauru, SP, Brazil.; 4 DDS, MSc, PhD, Associate Professor, Department of Pediatric Dentistry, Orthodontics and Community Health, Bauru School of Dentistry, University of São Paulo, Bauru, SP, Brazil.

**Keywords:** Malocclusion, angle class II, Severity

## Abstract

**Objectives:**

It is well known that the efficacy and the efficiency of a Class II malocclusion treatment are aspects closely related to the severity of the dental anteroposterior discrepancy. Even though, sample selection based on cephalometric variables without considering the severity of the occlusal anteroposterior discrepancy is still common in current papers. In some of them, when occlusal parameters are chosen, the severity is often neglected. The purpose of this study is to verify the importance given to the classification of Class II malocclusion, based on the criteria used for sample selection in a great number of papers published in the orthodontic journal with the highest impact factor.

**Material and Methods:**

A search was performed in PubMed database for full-text research papers referencing Class II malocclusion in the history of the American Journal of Orthodontics and Dentofacial Orthopedics (AJO-DO).

**Results:**

A total of 359 papers were retrieved, among which only 72 (20.06%) papers described the occlusal severity of the Class II malocclusion sample. In the other 287 (79.94%) papers that did not specify the anteroposterior discrepancy severity, description was considered to be crucial in 159 (55.40%) of them.

**Conclusions:**

Omission in describing the occlusal severity demands a cautious interpretation of 44.29% of the papers retrieved in this study.

## INTRODUCTION

**Figure 1:**
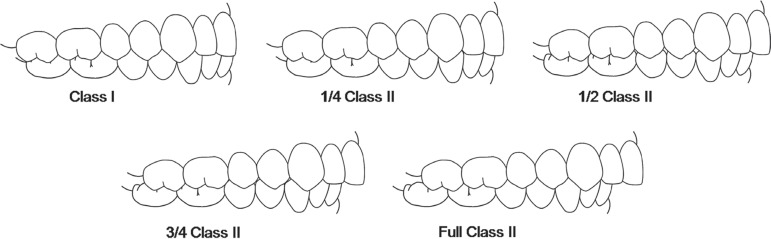
Illustration of a Class I anteroposterior relationship and increasing Class II malocclusion anteroposterior occlusal severities

Communication among practitioners was dramatically simplified when Angle^7^ (1899) first described the classification of malocclusion. By that time, he proposed only 3 categories in which malocclusions should be fitted in. Years after that, Andrews^4^ (1972) understood the necessity of a more complete classification as he proposed the six keys to normal occlusion and described a more precise classification of the anteroposterior occlusal discrepancy^5,6^ (Figure 1). This upgrade in the description of malocclusion not only facilitated comprehension of the problem but also gave Orthodontics a more scientific aspect. Currently, classification of Class II malocclusion is primarily based on these authors. Despite their effort to improve it, there is still a need for more details when describing the anteroposterior discrepancy^1,31^.

Recognition of occlusal malocclusion severity is important to determine the best treatment approach. The same malocclusion although with differing severity will be amenable to very different treatment protocols^11,20,23,28^. A full cusp Class II malocclusion, for example, requires more patient compliance in using removable orthodontic devices and more ability and experience of the orthodontist, than a ¼ cusp Class II malocclusion^22^. However, it is very unusual to find papers that clearly provide the occlusal discrepancy severity of the sample used. Additionally, the use of cephalometric variables is often more common than the occlusal parameters, although suggestion of including additional occlusal details has been made^24,38^.

Concerns about these omissions and the quality of the published studies is not a recent issue^24,30,36-38^. Research design, sample size and selection are the major source of bias in all studies assessed^38^. Therefore, the purposes of this study were to identify the importance given to the description of Class II malocclusion occlusal severity and to discuss its implications.

## MATERIAL AND METHODS

**Figure 2:**
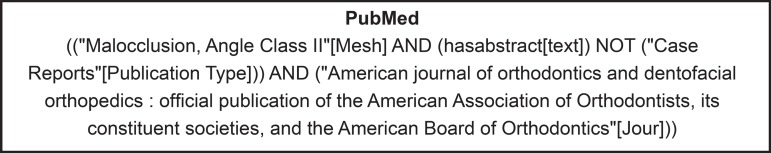
Database and method of search

In January 2008 a search was performed in PubMed database (Figure 2). The objective was to find research papers dealing with Class II malocclusion samples. Case reports were not included. For a more uniform search, only one journal was considered, the American Journal of Orthodontics and Dentofacial Orthopedics. This would minimize the odds of combining papers with different selection standards. There was no date limit for the search and every one of the 359 retrieved papers was analyzed.

The Material and Methods section of each paper was thoroughly read, and the criterion used for sample inclusion or exclusion was recorded. This sample selection criterion was considered only if it was placed under the Material and Methods section. When there was no Material and Methods section available, the whole paper was read. The goal was to understand the importance given to the malocclusion severity description.

The papers were divided based on the malocclusion report. The parameters used to describe the sample varied greatly. Some described malocclusion severity of the sample in a clear, precise way. Others used only occlusal features or only cephalometric variables or even both to describe the sample, but without specifying the occlusal severity. And yet, there were even those that did not mention any sort of severity parameter.

Use of the term "Angle Class II malocclusion" or similar, was considered as occlusal parameter, following the root of Angle’s classification. In cases that the criteria for sample selection were unclear, common sense was used to classify the paper in the most precise parameter possible.

Based on these data, the papers were ultimately divided as: "With Occlusal Severity Specification" and "Without Occlusal Severity Specification" (Table 1). The following terms were accepted as severity descriptors: mild, moderate, severe; complete, full unit, full cusp, cusp-to-cusp, half cusp, half cusp unit; edge-to-edge, end-to-end, end-on and the use of quarters or millimeters.

**Table 1:** Prevalence of Class II malocclusion occlusal severity specification

**Occlusal Severity Specification**		
**With/Without**	**Number of papers**	**Percentage**
		
With	72	20.06%
Without	287	79.94%

To be included in the "With Occlusal Severity Specification" category, the criteria had to be occlusal severity only, in a clear way, regardless of any other occlusal or cephalometric parameter. Therefore, the few papers that used primarily other parameters for sample selection, such as overjet or the ANB angle and secondarily also mentioned occlusal severity, were placed in other category.

When all papers were classified, their objectives were analyzed to verify if there was, indeed, a need for occlusal severity specification. The abstracts of those that did not specify the occlusal severity level were analyzed and separated according to the need of occlusal severity description based on the aim of the paper (Tables 2 and 3). In those papers concerning comparative studies, orthodontic device effects, treatment, protocol or technique effects, or investigation of Class II malocclusion characteristics, occlusal severity specification was considered mandatory. In reviews and researches, severity was not considered relevant. Papers that used different samples, such as Class I, were eliminated. Those were probably retrieved in the search because of the terms used by the authors as descriptors.

**Table 2:** Need for occlusal severity specification

**Papers Without Occlusal Severity Specification**	
**Considered necessary**	**Not considered necessary**
	
159 (55.40%)	128 (44.60%)

**Table 3:** Overall need for occlusal severity specification

**With**	**Without**	**Without**
**Severity Specification**	Severity Specification*	Severity Specification**
		
72 (20.06%)	128 (35.65%)	159 (44.29%)

* Occlusal severity specification was not considered crucial

** Occlusal severity specification was considered crucial

To understand the evolution of sample description, the papers were divided into four time intervals to easily demonstrate how much importance occlusal severity report has gained in the last years (Tables 4 and 5).

**Table 4:** Evolution of occlusal severity specification throughout time

**Group**	**With Specification**	**Without Specification**
		
Group 1 (1986-1992)	7 (10.14%)	62 (89.86%)
Group 2 (1993-1997)	16 (21.05%)	60 (78.95%)
Group 3 (1998-2002)	15 (15.00%)	85 (85.00%)
Group 4 (2003-2007)	34 (29.82%)	80 (70.18%)

**Table 5:** Need for occlusal severity specification throughout time

**Without Specification**		
**Group**	**Necessary**	**Not necessary**
		
Group 1 (1986-1992)	24 (38.71%)	38 (61.29%)
Group 2 (1993-1997)	39 (65.00%)	21 (35.00%)
Group 3 (1998-2002)	45 (52.94%)	40 (47.06%)
Group 4 (2003-2007)	51 (63.75%)	29 (36.25%)

## RESULTS

Among the 359 retrieved papers, 72 (20.06%) quoted the occlusal anteroposterior discrepancy severity amount and used it as a single parameter for sample selection (Table 1). The other 287 (79.94%) papers did not specify the occlusal malocclusion severity or, if so, it was only considered if other occlusal and cephalometric parameters had been satisfied. In the papers that did not specify the anteroposterior discrepancy severity, description was considered to be crucial in 159 (55.40%) (Table 2). Considering the total sample of 359 papers, severity description should have been mentioned in 44.29% of them (Table 3).

Concern in describing occlusal anteroposterior discrepancy severity increased in the latest years (Table 4). Between 2003 and 2007, occlusal severity specification represented 29.82% of the sample while between 1986 and 1992 it was of only 10.14% (Table 4). Conversely, omission of severity description in those papers where it was considered to be essential increased lately (Table 5). Between 1986 and 1992, this omission was of 38.71%, and increased to 63.75% between 2003 and 2007.

## DISCUSSION

There is a restless need for more clear, fair and accurate papers so conclusions can be extrapolated to the clinical practice. To identify if some basic elements methodically took part in the literature, a search was performed to check whether Class II malocclusion occlusal severity was appropriately described in the papers, when necessary. To work with a reasonable amount of representative high standard orthodontic papers, this search retrieved only those published in the AJO-DO because, according to the 2007 ISI Journal Citation Reports, this journal is the highest ranked orthodontic title, by number of citations and impact factor.

### Prevalence of Occlusal Severity Specification

Angle^7^ (1899) and Andrews^4^ (1972) used occlusal rather than cephalometric parameters to describe malocclusion of the teeth. Despite that, some of the latest orthodontic papers still classify malocclusion based only on cephalometric variables instead of using occlusal parameters. In some of them, part of the sample was rejected because it presented "dental but not skeletal Class II malocclusion"^32,34^.

Among the 359 papers retrieved in this search, only 72 (20%) specified the occlusal severity of the sample, regardless of any other occlusal or cephalometric parameter (Table 1). This demonstrates the little importance given to dental relationships, which are the most important characteristics to be corrected in the great majority of orthodontic cases^27^. This probably happened because after development of cephalometrics there was an emphasis on the cephalometric characteristics of the malocclusion, placing dental relationships on a secondary level^15^. Along with the cephalometric characteristics, emphasis was given on the skeletal components of malocclusion and this has been the tendency throughout time^13,29^. However, it is usually not the skeletal characteristics of a Class II malocclusion that primarily determine how it should be treated but, rather, the dentoalveolar characteristics^19^. In addition, it has also been shown that the cephalometric variables will influence the esthetic prognosis but not the treatment occlusal success rate^21^.

Some papers have selected skeletal Class II malocclusion subjects based exclusively on cephalometric parameters^15,18^. There is a great deficiency in this procedure because patients can present a normal occlusion despite great basal bone cephalometric anteroposterior discrepancy^42^. Therefore, whenever the effects of a certain appliance in the correction of Class II malocclusion are evaluated, it is crucial that the occlusal anteroposterior discrepancy should be clearly measured. This procedure not only clarifies the sample characteristics but also best describes the treatment difficulty of the case.

Overjet was also frequently used as description for anteroposterior discrepancy. Sometimes it was used as the only parameter as if it was only present in Class II malocclusions. Overjet is very influenced by labial inclination of anterior teeth. The presence of diastemas can also significantly increase it and it is very possible to have a Class I malocclusion with increased overjet. In these cases, the maxillary incisors may be severely labially tipped associated or not with diastemas and the mandibular anterior teeth may be crowded^3,12,26^. This does not require great mechanical effort to correct the anteroposterior discrepancy because the posterior teeth are in a Class I relationship^42^. Therefore, this parameter is by no means enough to describe Class II malocclusion severity.

Deficiency in severity description was evident in some papers were malocclusion was described as "borderline subjects"^9^ or "Class II incisal relationship"^16,35^ or "most Class II subjects"^40^ or "there was a preponderance of Class II malocclusions"^33^ or "all Class II patients"^28^ or "the profile had a Class II appearance"^8^ or "…this study was not treatment of *any* Class II malocclusion; it was a study of the orthodontic treatment of *difficul* *t* Class II malocclusions…"^17^ (Italics from the author). These descriptors are very indistinct and do not allow a precise estimation of the amount of Class II anteroposterior discrepancy.

It was also observed that usually the experimental groups followed rigid occlusal anteroposterior discrepancy criteria while the control groups did not^25^. Therefore, results of these comparisons could be compromised. Omission in describing Class II malocclusion severity could explain why there are sometimes contrasting results. While some authors report significant effects, others fail in demonstrating them. Therefore, basic questions remain unanswered^38^.

### Studies that Demanded Occlusal Severity Specification

Papers that did not specify the severity level were analyzed whether occlusal severity specification was mandatory based on the aim of the study. Among those classified as "Without Occlusal Severity Specification", in 159 papers (55.40%) severity specification was judged to be crucial. This means that the results and conclusions of these works could be compromised by the unspecific anteroposterior severity description. As it is known, comparative studies and Class II investigations need matched samples to avoid bias. Furthermore, if a device or technique is being tested in a sample with mild severity, results naturally tend to praise the system being tested without considering the simplicity to correct the Class II malocclusion (Tables 2 and 3).

### Occlusal Severity Specification over the Years

The results demonstrated that occlusal severity description has gained some attention through the years. Specification increased three times from 1986 to 2007. It seems that concerns in specifying Class II malocclusion severity reflects the improvement in malocclusion classification developed by Andrews, with his paper "The six keys to normal occlusion", in 1972^4^. His textbook also illustrates how cases from the American Board progressively improved at the end of treatment in the 1960's, 1970's and 1980's^6^. This improvement is certainly due to the consideration and attention on the final occlusal aspects of the cases to be judged, since the mission of the American Board of Orthodontics is to establish and maintain the highest standards of clinical excellence by evaluating clinical competence^39^. As concerns with detailed finishing increased, investigators and clinicians realized that greater specification of malocclusion severity, especially Class II malocclusion anteroposterior discrepancy, was necessary to satisfactorily describe treatment difficulty. However, although there have been an increasing number of papers describing Class II malocclusion occlusal severity (Table 4), the percentage of papers without specification in which it was mandatory increased (Table 5). This shows that the importance of malocclusion occlusal severity has been underestimated.

It must been understood that Class II malocclusion occlusal severity specification is correlated to treatment plan and time, and to the mechanical difficulty in handling the malocclusion, and therefore it has to be precisely described^2,10,14,21,22,41^. Class II malocclusion occlusal severity features should be more thoroughly described in scientific papers to provide a better understanding of treatment difficulties of this malocclusion.

## CONCLUSIONS

Class II malocclusion occlusal severity description is a very important characteristic and has to be specified in the great majority of Orthodontic papers. This parameter is well-known and very simple to understand and to use as a classification. Despite the importance and the simplicity of occlusal severity specification, it has not been systematically used. Consequently, the results of some papers should be cautiously interpreted.
